# Genome-Wide Characterization and Expression Analysis of *CAMTA* Gene Family Under Salt Stress in *Cucurbita moschata* and *Cucurbita maxima*

**DOI:** 10.3389/fgene.2021.647339

**Published:** 2021-06-17

**Authors:** Jingping Yuan, Changwei Shen, Bihua Chen, Aimin Shen, Xinzheng Li

**Affiliations:** ^1^School of Horticulture and Landscape Architecture, Henan Institute of Science and Technology, Xinxiang, China; ^2^Henan Engineering Research Center of the Development and Utilization of Characteristic Horticultural Plants, Xinxiang, China; ^3^School of Resources and Environmental Sciences, Henan Institute of Science and Technology, Xinxiang, China; ^4^Zhengzhou Vegetable Research Institute (ZVRI), Zhengzhou, China

**Keywords:** *Cucurbita* linn, *CAMTA*, genome-wide identification, salt stress, expression analysis

## Abstract

*Cucurbita* Linn. vegetables have a long history of cultivation and have been cultivated all over the world. With the increasing area of saline–alkali soil, *Cucurbita* Linn. is affected by salt stress, and calmodulin-binding transcription activator (CAMTA) is known for its important biological functions. Although the CAMTA gene family has been identified in several species, there is no comprehensive analysis on *Cucurbita* species. In this study, we analyzed the genome of *Cucurbita maxima* and *Cucurbita moschata*. Five *C. moschata* calmodulin-binding transcription activators (*CmoCAMTAs*) and six *C. maxima* calmodulin-binding transcription activators (*CmaCAMTAs*) were identified, and they were divided into three subfamilies (Subfamilies I, II, and III) based on the sequence identity of amino acids. CAMTAs from the same subfamily usually have similar exon–intron distribution and conserved domains (CG-1, TIG, IQ, and Ank_2). Chromosome localization analysis showed that *CmoCAMTAs* and *CmaCAMTAs* were unevenly distributed across four and five out of 21 chromosomes, respectively. There were a total of three duplicate gene pairs, and all of which had experienced segmental duplication events. The transcriptional profiles of *CmoCAMTAs* and *CmaCAMTAs* in roots, stems, leaves, and fruits showed that these *CAMTAs* have tissue specificity. *Cis*-acting elements analysis showed that most of *CmoCAMTAs* and *CmaCAMTAs* responded to salt stress. By analyzing the transcriptional profiles of *CmoCAMTAs* and *CmaCAMTAs* under salt stress, it was shown that both *C. moschata* and *C. maxima* shared similarities against salt tolerance and that it is likely to contribute to the development of these species. Finally, quantitative real-time polymerase chain reaction (qRT-PCR) further demonstrated the key role of *CmoCAMTAs* and *CmaCAMTAs* under salt stress. This study provided a theoretical basis for studying the function and mechanism of *CAMTAs* in *Cucurbita* Linn.

## Introduction

Environmental conditions are highly important to the growth and productivity of plants. However, the adverse environment caused by biotic and abiotic stresses typically allows plants to cope with it by developing or activating some mechanisms (Yamniuk and Vogel, 2004). One of the prime research priorities for scientists in recent years has been to clarify the mechanism of plant stress response. For this purpose, several genes and pathways have been identified (Büyük et al., 2016). Research on plant transcription factors (TFs), for example, has been rapidly increased in the regulation of stress-related genes. With this development, the identification of TFs in plants at the whole-genome stage has become an increasingly popular source of research and is necessary for understanding the plant stress response (Yamniuk and Vogel, 2004; Büyük et al., 2016; Inal et al., 2017). TFs play important roles in cell and non-cell signal transduction by interacting with *cis*-elements (Wei et al., 2017).

As the secondary messenger of eukaryotes, Ca^2+^ ions play important roles in gene transcription and intracellular signal transduction. At present, many sensor proteins, such as calmodulin (CaM), are responsive to Ca^2+^ concentration changes inside and outside cells (İlhan et al., 2018). Calmodulin-binding transcription activator (CAMTA) family is called a rapid stress response element when screening calmodulin-binding protein (Min et al., 2009; Pant et al., 2018). Based on functional differentiation, CAMTA included a variety of functional domains: (1) CG-1 DNA-binding domain at the N terminal, which includes nuclear localization signal; (2) ankyrin repeats, which are responsive to the mediated interaction of protein–protein; (3) calmodulin binding domain (CaMBD), which includes different numbers of IQ motifs (IQXXXRGXXXR). These domains combine with CaM in a Ca^2+^-independent way (Bouché et al., 2002; Kaplan et al., 2006; Finkler et al., 2007; Yang et al., 2012).

The *CAMTA* gene was first reported in tobacco (Yang and Poovaiah, 2000). Since then, the *CAMTA* gene family has been identified in many species, for instance, 6, 7, 10, 9, 15, 18, and 7 genes were found in *Arabidopsis thaliana*, *Solanum lycopersicum*, *Vitis vinifera*, *Zea mays*, *Glycine max*, *Brassica napus*, and *Populus trichocarpa*, respectively (Bouché et al., 2002; Yang et al., 2012; Shangguan et al., 2014; Yue et al., 2015; Wang et al., 2015; Rahman et al., 2016a; Wei et al., 2017). Related literature showed that CAMTA TFs were involved in responses to a variety of stresses, including drought stress, heat stress, cold stress, salt stress, ultraviolet ray, wound stress, abscisic acid, and salicylic acid (Yang et al., 2012, 2015; Pandey et al., 2013; Zhao et al., 2013; Shangguan et al., 2014; Yue et al., 2015; Rahman et al., 2016a; Wei et al., 2017).

In *A. thaliana*, *AtCAMTA1* regulated drought stress by acting on the *cis*-acting elements of several stress-responsive genes, such as *RD26*, *ERD7*, *RAB18*, *LTPs*, *COR78*, *CBF1*, and *HSPs* (Pandey et al., 2013). At the same time, *AtCAMTA1* mutants showed lower drought resistance than the wild type in *A. thaliana* (Pandey et al., 2013). In addition, *AtCAMTA3* regulated the defense response of pathogens by activating *EDS1*-mediated SA signals (Du et al., 2009), and it could also directly regulate *NDR1* and *EIN3* to participate in ethylene-induced senescence (Nie et al., 2012). *A. thaliana CAMTA1*–*CAMTA3* double mutants were more sensitive to cold stress (Doherty et al., 2009). The *CAMTA* genes in *Phaseolus vulgaris* have been identified, and all *PvulCAMTA* genes were targeted by miRNAs, which play a role in the response mechanism of salt stress (Büyük et al., 2019).

*Cucurbita* Linn. vegetables have a long history of cultivation and are cultivated all over the world. In recent years, *Cucurbita* Linn. has been paid more and more attention as a yellow-green vegetable with healthcare benefits (Henz et al., 2020; Ibeh et al., 2020). *Cucurbita* Linn. seeds and pulp have high nutritional value and are suitable for deep processing. However, with the increasing saline–alkali soil area, it is particularly important to study the salt-resistant mechanism and screen salt-tolerant varieties of *Cucurbita* Linn. as a non-salt vegetable (Zhao, 2006). *C. maxima* and *C. moschata* are the two main cultivated species of *Cucurbita* Linn.

Many CAMTAs have been identified in different plants through the whole-genome identification method, but there is no report in *Cucurbita* Linn. (Wang et al., 2015; Wei et al., 2017; İlhan et al., 2018). The main purpose of this study is to identify and characterize the *CAMTA* gene in *C. maxima* and *C. moschata* and to explore their crucial role under salt stress. In this study, several bioinformatics tools were used to analyze the number, distribution, classification, gene structure, protein structure, gene duplication, and evolution of *CAMTA* genes in *C. maxima* and *C. moschata*. Moreover, to validate the function of the *CAMTA* genes under salt stress, we also performed RNA-seq and qRT-PCR analyses. The result of this study is of great significance to the genetic improvement of salt-tolerant varieties of *Cucurbita* Linn.

## Materials and Methods

### Identification and Characterization of *CAMTAs* in *C. moschata* and *C. maxima*

To identify the CAMTAs in *Cucurbita* Linn., we downloaded the *C. moschata* and *C. maxima* genomes from the *Cucurbit* genomics database (CuGenDB^1^) (Sun et al., 2017). The proteins of six *A. thaliana CAMTAs* (*AtCAMT1–AtCAMT6*) were downloaded from the NCBI database^2^ by using their gene IDs from previous literature (Zhang et al., 2019), and they were used as search queries against the *Cucurbit* genomics database by BLASTP. The E-value cutoff was set up at a threshold of 1 × e^–10^, and the protein sequences with less than 70% of the corresponding *A. thalian*a were eliminated from the study. In addition, we used CD-Search^3^ and SMART^4^ to verify the different CAMTA domains such as the IPT/TIG, IQ motifs, ankyrin repeats, and CG-1 DNA-binding domain. After removing the false-positive genes, the remaining genes were termed *C. moschata CAMTAs* (*CmoCAMTAs*) and *C. maxima CAMTA* (*CmaCAMTAs*). The information on the coding sequence and protein sequence of *CmoCAMTA* and *CmaCAMTA* is listed in Supplementary Table 1.

The physicochemical characteristics of *CmoCAMTAs* and *CmaCAMTAs* include the theoretical isoelectric point (*pI*), the length of amino acids (aa), and theoretical molecular weight (MW). All of them were analyzed by ExPASy^5^. The subcellular locations of *CmoCAMTA* and *CmaCAMTA* were predicted by Plant-mPLoc (Chou and Shen, 2010).

### Construction of Phylogenetic Tree

To construct the unrooted evolutionary tree of CAMTAs from *C. moschata* and *C. maxima*, CmoCAMTA and CmaCAMTA protein sequences were downloaded from the *Cucurbit* genomics database, and the tree was constructed with the help of MEGA 7.0 (Sudhir et al., 2016) using the neighbor-joining (NJ) method. The parameters were set as follows: completed deletion, Poisson model, and a branch tree cutoff value of 80%. Also, MEGA 7.0 was used to analyze the phylogenetic relationships of CAMTA in *A. thaliana*, *C. moschata*, and *C. maxima*.

### Structure Analysis of CAMTAs From *C. moschata* and *C. maxima*

To explore the structural characteristics of *CAMTAs* in *C. moschata* and *C. maxima*, the genomic DNA and corresponding cDNA sequences were downloaded from the *Cucurbit* genomics database. The intron-exon structure pattern was mapped by Gene Structure Display Server (GSDS^6^) (Hu et al., 2015).

To further analyze the conserved motif of CAMTAs in *C. moschata* and *C. maxima*, the protein sequences of CmoCAMTAs and CmaCAMTAs were used. The conserved motifs were presented on the Multiple Expectation Maximization or Motif Elicitation (MEME^7^) (Bailey and Elkan, 1995), while the LOGOs (Supplementary Figure 1) of motifs can also be presented through MEME.

To analyze the conserved domain of CAMTA protein in *C. moschata* and *C. maxima*, the protein sequences of CmoCAMTAs and CmaCAMTAs were used. The location information of the conserved domain was extracted from Batch Web CD-Search Tool^8^ (Marchler-Bauer et al., 2017), and the Simple BioSequence Viewer (Chen et al., 2020) in TBtools was finally used for visualization. The information about these conserved domains in CAMTA proteins from *C. moschata* and *C. maxima* is listed in Supplementary Table 2.

### Gene Duplication of *CAMTAs* in *C. moschata* and *C. maxima*

Information on *CmoCAMTA* and *CmaCAMTA* genes, including the length of the genes on the chromosome, the starting position, and the terminal position of the genes on the chromosome, was investigated in the *Cucurbit* genomics database. The chromosomal locations of the *CmoCAMTAs* and *CmaCAMTAs* were mapped by visualization tools^9^.

To identify gene duplication, all *CmoCAMTAs* used Local Blast for the blastn program, and when the nucleotide sequence identity was greater than 85%, the *E*-value was less than 1 × e^–10^, and the gene alignment coverage was greater than 0.75; the two genes were considered to be a duplicated gene pair (Yuan et al., 2019). In addition, two genes were separated by one or several genes, as long as the distance between the two genes was less than 100 kb; it was called tandemly duplicated genes (Wang et al., 2010). The synonymous substitution ratio (*Ks*) was calculated on a *Ka*/*Ks* calculator according to Gojo-bori and Nei’s previous method (Zhang et al., 2006). In order to remove the saturation of substitutions, we discard gene pairs with *Ks* > 2.0 (Blanc and Wolfe, 2004; Li et al., 2014). The divergence time (*T*) of the duplicated genes was calculated based on *T* = *Ks*/2λ × 10^–6^ million years ago (Mya), λ = 1.5 × 10^–8^ (Emanuelsson et al., 2000).

### Extraction of *Cucurbita* Linn. *CAMTA* Promoter Sequence and Analysis of *Cis-*Acting Elements

To analyze the salt stress-related *cis*-acting elements of *Cucurbita* Linn., promoter sequences (2,000 bp before the start codon) were extracted from the *Cucurbit* genomics database. These sequences were analyzed on the PlantCARE program^10^ and finally displayed by the Simple BioSequence Viewer in TBtools (Chen et al., 2020).

### Expression Profiles of *Cucurbita* Linn. *CAMTAs* in RNA-Seq

To analyze the tissue expression characteristics of *Cucurbita* Linn. *CAMTAs*, the transcriptional profiles of *C. moschata* and *C. maxima* in root, stem, leaf, and fruit were analyzed according to the previously published transcriptome data (BioProject: PRJNA385310) (Sun et al., 2017). In this study, “*Rifu*” in *C. moschata* and “*Rimu*” in *C. maxima* were used as research materials.

To analyze the response of *Cucurbita* Linn. to salt stress, the transcript profiles of *CAMTAs* in leaf veins and leaf mesophylls were analyzed according to the transcriptome data (BioProject: PRJNA464060) (Niu et al., 2018). “N12” in *C. moschata* and “N15” in *C. maxima* were used as research materials.

### Materials and Experimental Treatment

In this study, “*Baimi 9*” from *C. moschata* and “*Beiguan*” from *C. maxima* were used as research materials to analyze the expression of *CAMTAs* under salt stress. The seeds were provided by the pumpkin team of the School of Horticulture and Landscape Architecture, Henan Institute of Science and Technology. The seeds were first sown in a tray with a matrix meteorite (3:1) mixture and then placed in a plant growth chamber for cultivation. The artificial growth conditions were set as follows: daytime temperature of 25°C, 16 h of light, light intensity of 350 μmol/m^2^/s, night temperature of 16°C, and a relative humidity of 65%. When the seedlings have grown to 2 months, healthy and neat seedlings were selected and cultured in Hoagland’s solution at pH 6.5. After 5 days of adaptation, 50 healthy and consistent seedlings of each variety were selected for NaCl treatment (the concentration of NaCl was 75 mM), and the remaining 50 seedlings were used as control. The veins and mesophyll were collected after 12 h of salt treatment, and each treatment had three independent biological replicates, and 10 seedlings per biological replicate were selected for mixed sampling. All samples were frozen in liquid nitrogen and stored at −70°C for quantitative real-time PCR (qRT-PCR) analysis.

### qRT-PCR Analysis

The control and salt-treated samples were taken from the freezer at −70°C and then fully ground with liquid nitrogen in the molding machine. The RNA was extracted by RNA-Solv^@^ reagent (Omega) and reverse-transcribed into cDNA with PrimeScript^TM^ RT Master Mix (TaKaRa) after DNase treatment, which was used as a template for qRT-PCR determination. The primers of *CmoCAMTAs*, *CmaCAMTAs*, and internal reference gene (β-actin) were first designed on Prime premier 6.0 and then blasted in the *Cucurbit* genomics database to verify the specificity of primers (Supplementary Table 3). Finally, the specificity of the primers was verified by the melting curve on Applied Biosystems 7,500. The reaction system included 10 μl of SYBR Green I, 2 μl of cDNA template, 0.4 μl of ROX dye II, 0.4 μl of primers, and 6.8 μl of ddH_2_O. The reaction conditions were set as follows: 95°C pre-denaturation for 30 s, 95°C for 5 s, and 60°C for 34 s (40 cycles). The melting curve was 95°C for 15 s, 60°C for 60 s, and 95°C for 15 s. Each sample was performed with three technical replicates, and the data were analyzed with the 2^–ΔΔ*Ct*^ method (Livak and Schmittgen, 2001) and presented by HeatMap in TBtools (Chen et al., 2020).

## Results

### Identification and Characterization of *CmoCAMTAs* and *CmaCAMTAs*

Through the BLASTP program of six AtCAMTA proteins in the *Cucurbit* genomics database and a series of false positives and the same gene deletion steps, five *CmoCAMTAs* and six *CmaCAMTAs* were identified. According to their distribution on chromosomes (from the first chromosome to the last chromosome, from the top position to the end position of one chromosome), these genes were named *CmoCAMTA1*-*CmoCAMTA5* and *CmaCAMTA1*-*CmaCAMTA6*, respectively.

The physical and chemical characteristics of five CmoCAMTAs and six CmaCAMTAs are listed in Table 1. The coding region of *CmoCAMTAs* ranged from 2,745 (*CmoCAMTA4*) to 3,270 bp (*CmoCAMTA5*), and the corresponding translated amino acids ranged from 914 to 1,089 aa (Table 1). Their theoretical molecular weight (MW) and theoretical isoelectric point (*pI*) were 103.36 (CmoCAMTA4) to 121.38 kDa (CmoCAMTA5) and 5.69 (CmoCAMTA5) to 7.7 (CmoCAMTA2), respectively. Similarly, the coding region of CmaCAMTAs ranged from 2,748 (*CmaCAMTA5*) to 3,273 bp (*CmaCAMTA6*), and the corresponding translated amino acids ranged from 915 to 1,090 aa (Table 1). Their theoretical MW and *pI* were 103.44 (CmaCAMTA5) to 121.64 kDa (CmaCAMTA3) and 5.66 (CmaCAMTA3) to 7.71 (CmaCAMTA5), respectively. Both CmoCAMTAs and CmaCAMTAs revealed diversity in the coding region, amino acid sequence, and MW and had low isoelectric points. Subcellular localization prediction analysis showed that all CmoCAMTAs and CmaCAMTAs were localized to the nucleus.

**TABLE 1:** Physical and chemical characteristics of the 5 CmoCAMTAs and 6 CmaCAMTAs.

Gene ID	Gene name	Cmo_Chr *^1^	Start*^2^	End*^3^	ORF length	AA*^4^	pI*^5^	Mw*^6^ (Da)	Loc*^7^
					(bp)				
**CmoCh08G001800.1**	***CmoCAMTA1***	8	1057956	1072099	1701	937	5.82	105321.74	Nucleus.
**CmoCh19G002740.1**	***CmoCAMTA5***	19	2018145	2018184	945	1089	5.69	121380.57	Nucleus.
**CmoCh17G003490.1**	***CmoCAMTA4***	17	2121934	2122009	1017	914	7.21	103358.58	Nucleus.
**CmoCh14G016000.1**	***CmoCAMTA3***	14	12669848	12669887	723	956	6.56	106714.33	Nucleus.
**CmoCh08G012360.1**	***CmoCAMTA2***	8	7820471	7820546	900	919	7.7	104643.86	Nucleus.
**CmaCh08G001770.1**	***CmaCAMTA1***	8	990830	990839	1392	981	6.14	110594.34	Nucleus.
**CmaCh19G002540.1**	***CmaCAMTA6***	19	1819878	1819917	1218	1090	5.7	121207.17	Nucleus.
**CmaCh17G003580.1**	***CmaCAMTA5***	17	1985179	1985254	1134	915	7.71	103436.72	Nucleus.
**CmaCh08G012660.1**	***CmaCAMTA2***	8	7703880	7703955	1110	917	6.88	104375.36	Nucleus.
**CmaCh11G016500.1**	***CmaCAMTA3***	11	10856597	10856636	1362	1091	5.66	121644.44	Nucleus.
**CmaCh14G015650.1**	***CmaCAMTA4***	14	11743952	11743991	993	963	7.21	107511.62	Nucleus.

Information on including their chromosomal distribution, their start and the end positions on the chromosomes, nucleic acid sequence and amino acid sequence were extracted from Cucurbit genomics database, and all the data in the table is predicted or theoretical.

*1 Cmo_Chr, The name of the CAMTA chromosome corresponding to the gene.

*2 Start, Predicted starting position of mRNA.

*3 End, Predicted termination position of mRNA.

*4 AA, Amino acid number in CAMTA protein sequences.

*5 pI, Theoretical Isoelectric point.

*6 MW, Molecular weight (Mw) predicted by ExPASy (http://web.expasy.org/tools/).

*7 Loc, Subcellular location of the CAMTA proteins predicted by Plant-mPLoc.

### Phylogenetic Relationship of CAMTAs in *C. moschata*, *C. maxima*, and *A. thaliana*

**FIGURE 1:**
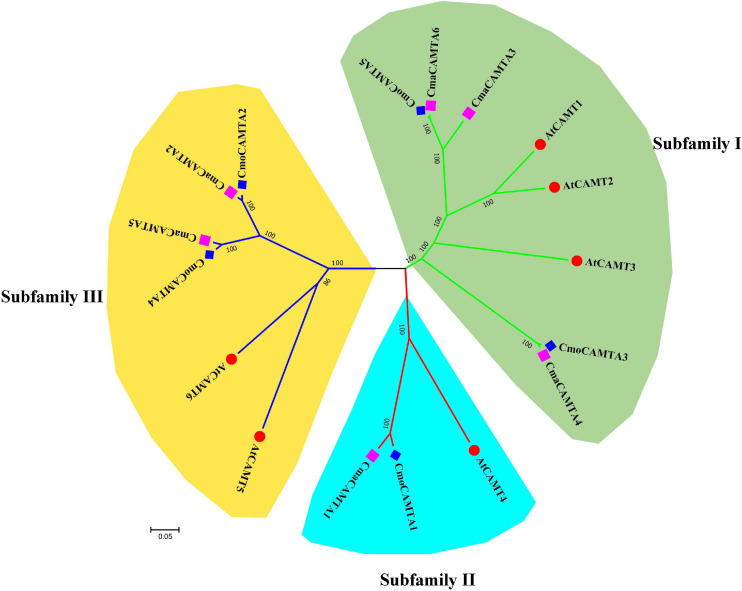
The phylogeny tree of five CmoCAMTAs, six CmaCAMTAs, and six AtCAMTAs. It was constructed by the neighbor-joining (NJ) method with 1,000 bootstrap replicates, and the cutoff value of the condensed tree was 80%. Each subfamily was highlighted with a specific background color.

According to the identity of amino acid sequence, five CmoCAMTAs, six CmaCAMTAs, and six AtCAMTAs were used to construct an unrooted evolutionary tree. As shown in Figure 1, these genes were divided into three subfamilies (Subfamilies I, II, and III). Subfamily I contained the most (eight) members, subfamily II contained the least (three) members; the remaining six proteins belong to Subfamily III. Each subfamily contained CmoCAMTA, CmaCAMTA, and *A. thaliana* CAMTA (Figure 1).

### Gene Structures of *CAMTAs* in *C. moschata* and *C. maxima*

**FIGURE 2:**
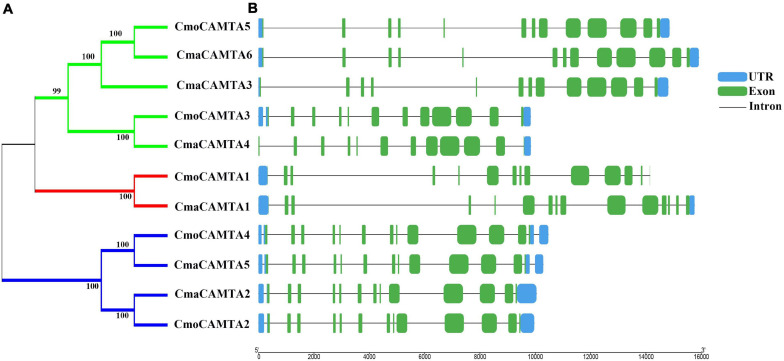
Classification and exon–intron distribution of five *CmoCAMTAs* and six *CmaCAMTAs.* **(A)** The phylogeny tree of five *CmoCAMTAs* and six *CmaCAMTAs.* It was constructed by the neighbor-joining (NJ) method with 1,000 bootstrap replicates, and the cutoff value of the condensed tree was 80%. **(B)** The exon-intron distribution of five *CmoCAMTAs* and six *CmaCAMTAs.* The GSDS was used, and the exons, introns, and untranslated regions (UTRs) were indicated with green boxes, blue boxes, and gray lines, respectively. The length of the introns and exons can be estimated based on the bottom scale.

By analyzing the intron–exon structure pattern of 11 *Cucurbita* Linn. *CAMTAs* (five *CmoCAMTAs* and six *CmaCAMTAs*), it showed that all *CAMTAs* contained 12–14 exons (Figure 2B). Some genes in the same branch contained similar structural features, such as *CmoCAMTA5*, *CmaCAMTA6*, and *CmaCAMTA3* contained 12 exons and similar intron lengths (Figures 2A,B). In addition, *CmoCAMTA4* as well as *CmaCAMTA5* and *CmoCAMTA2* as well as *CmaCAMTA2* also contained 12 and 13 exons with similar intron length, respectively (Figure 2B).

### Motif Composition and Conserved Domain of Five CmoCAMTAs and Six CmaCAMTAs

**FIGURE 3:**
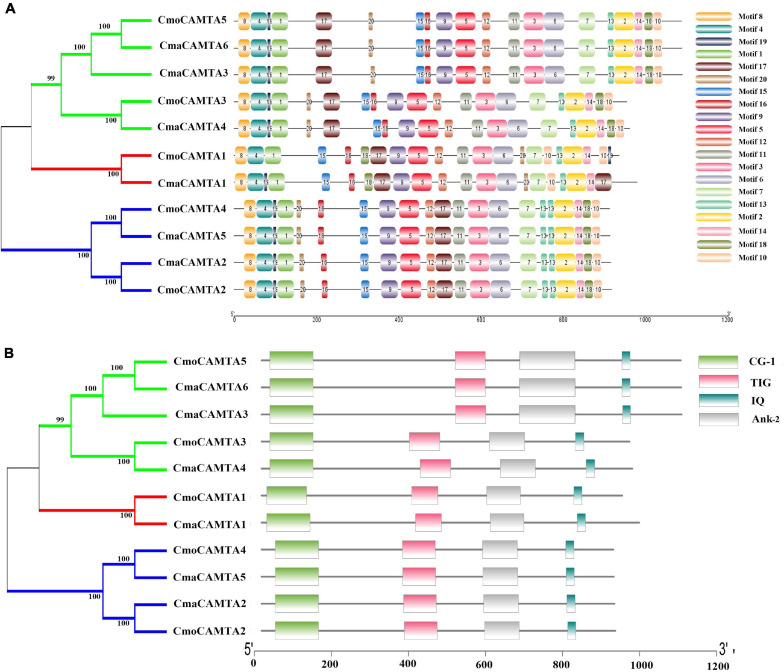
Conserved motifs and conserved domains of five CmoCAMTAs and six CmaCAMTAs. **(A)** Conserved motifs in five CmoCAMTAs and six CmaCAMTAs. Motif 1 to motif 10 represented different motifs, and they were represented by different color boxes on the right. **(B)** Conserved domains in five CmoCAMTAs and six CmaCAMTAs. CG-1, CG-1 domains. TIG, IPT/TIG domain. IQ, including the conserved sequences of Ile and Gln, is a calmodulin-binding motif. Ank_2, ankyrin repeats.

Motif analysis of CmaCAMTA and CmoCAMTA proteins indicated that motif 1 to motif 20 existed in all CAMTA proteins, but the CAMTA proteins in different subfamilies usually had different motif positions, such as motif 17, motif 15, and motif 16 (Figure 3A). CAMTA proteins in the same branches were similar, such as all CAMTA proteins in Subfamily III (Figure 3A). Conserved domain analysis showed that each CAMTA protein contained CG-1, TIG, IQ, and Ank_2 domains (Figure 3B). Detailed information about conserved domains is listed in Supplementary Table 2.

A comprehensive analysis of the motif and conserved domains of CmaCAMTA and CmoCAMTA proteins revealed that motif 1, motif 4, and part of motif 8 constituted the CG-1 domain; part of motif 9 and motif 5 constituted the TIG domain; motif 11, motif 3, and motif 6 constituted the Ank_2 domain; motif 2 constituted the IQ motif (Figures 3A,B). It is hypothesized that, based on the above study, most of them shared conserved structure like other CAMTAs in different species, and they are likely to respond to certain other stresses and stimulus signals.

### Distribution and Gene Duplication of Five *CmoCAMTAs* and Six *CmaCAMTAs*

**FIGURE 4:**
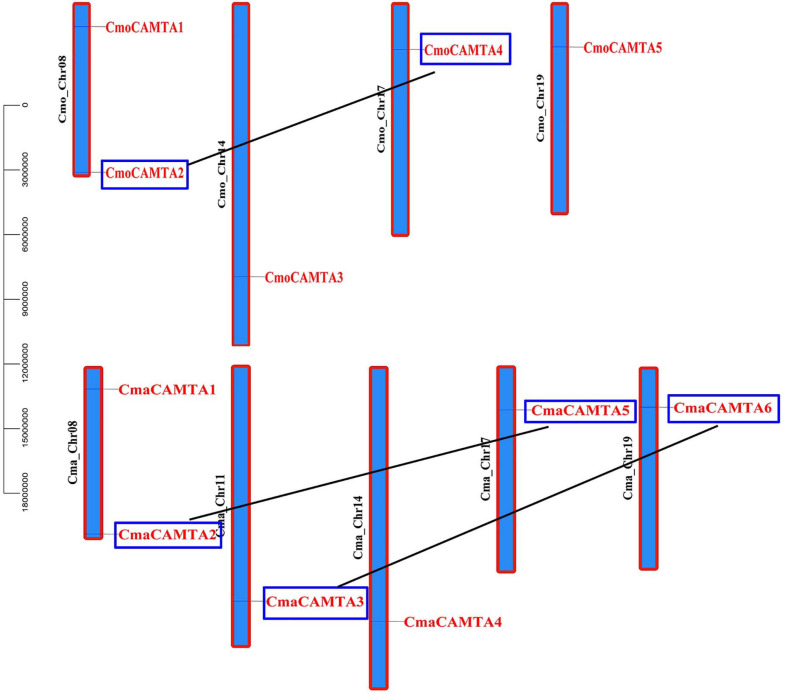
The distribution and duplication events of five *CmoCAMTAs* and six *CmaCAMTAs* on the chromosome. The location of these genes on the chromosome was visualized using the visualization tools. The duplicated gene pairs were indicated with blue boxes and connected by black lines.

To predict the location of *CmaCAMTAs* and *CmaCAMTAs* on the chromosomes, the start position of *CmaCAMTAs* and *CmaCAMTAs* and the length of the corresponding chromosomes were analyzed. The results showed that five *CmoCAMTAs* and six *CmaCAMTAs* were distributed across four (Cmo_Chr08, Cmo_Chr14, Cmo_Chr17, and Cmo_Chr19) and five (Cma_Chr08, Cma_Chr11, Cma_Chr14, Cma_Chr17, and Cma_Chr19) out of 21 chromosomes, respectively (Figure 4). In addition, three duplicated gene pairs (CmoCAMTA4_CmoCAMTA2, CmaCAMTA6_CmaCAMTA3, and CmaCAMTA5_CmaCAMTA2) were found in *Cucurbita* Linn. *Ka*/*Ks* indicated that the duplicated gene pairs had diverged at 8.62–9.64 million years ago (Mya) (Supplementary Table 4).

### *Cis*-Acting Elements in Five *CmoCAMTA* and Six *CmaCAMTA* Promoters

The *cis*-acting elements are specific motifs that exist in the promoter region of a gene sequence that regulates gene transcription. For instance, TGA-element, G-box, TGACG-motif, ABRE, GT1-motif, and MBS were all related to salt stress (Yamniuk and Vogel, 2004; Saeediazar et al., 2014). To explore whether *CmoCAMTAs* and *CmaCAMTAs* were involved in salt stress, we predicted and analyzed the *cis*-acting elements of these gene promoters. Details information about *cis*-acting elements are listed in Supplementary Table 5.

**FIGURE 5:**
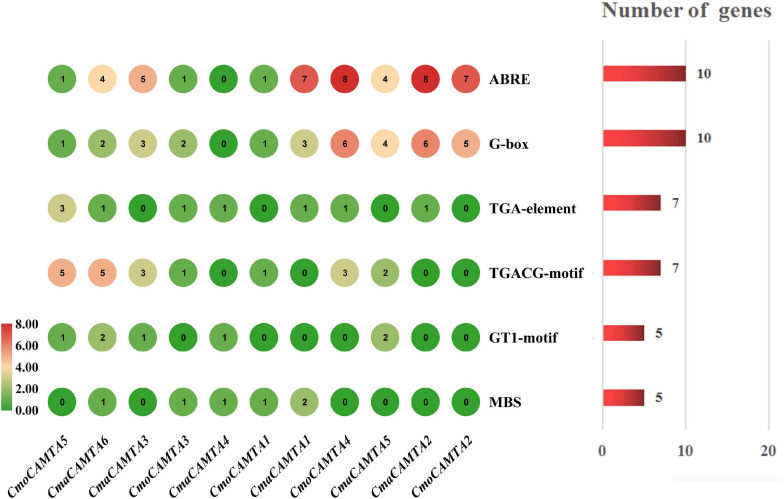
C*is*-acting elements in five *CmoCAMTAs* and six *CmaCAMTAs* promoters. The *cis*-acting elements were predicted by the PlantCARE program and displayed with the Simple BioSequence Viewer in TBtools. The circle represented the number of specific *cis*-acting elements per gene. The chart and number on the right indicated the number of genes corresponding to the specific *cis*-acting element.

The prediction results showed that *CmoCAMTA5* and C*maCAMTA6* promoters contained the highest number of (five) TGACG-motif (Figure 5). *CmaCAMTA6*, *CmaCAMTA3*, *CmaCAMTA1*, *CmoCAMTA4*, *CmaCAMTA5*, *CmaCAMTA2*, and *CmoCAMTA2* contained a higher number of (four to eight) ABRE compared with other gene promoters. At the same time, *CmoCAMTA4*, *CmaCAMTA5*, *CmaCAMTA2*, and *CmoCAMTA2* contain a higher number of (four to six) G-box than other genes (Figure 5). ABRE and G-box were presented in 10 of 11 *Cucurbita* Linn. CAMTA gene promoters (Figure 5), which fully reflect the response of *CmoCAMTAs* and *CmaCAMTAs* to salt stress.

### The Expression Profile of *CmoCAMTAs* and *CmaCAMTAs* in Different Tissues

**FIGURE 6:**
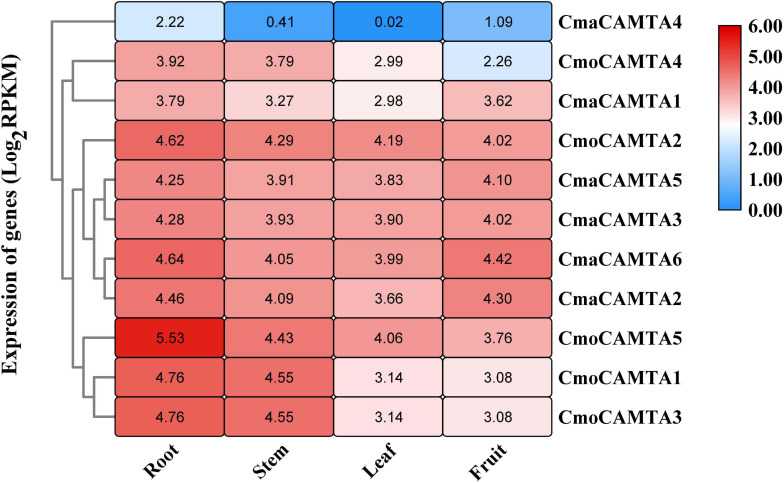
Heat map and hierarchical clustering of five *CmoCAMTAs* and six *CmaCAMTAs* in the root, stem, leaf, and fruit. All data and the bar on the right of the heat map were standardized by Log_2_ (RPKM).

By analyzing the expression profile of *CmoCAMTAs* and *CmaCAMTAs* in root, stem, leaf, and fruit, the results showed that except for *CmaCAMTA4*, other genes had higher expression profiles (Figure 6). In addition, the expression of all *CmoCAMTAs* and *CmaCAMTAs* in roots was higher than that in stem, leaf, and fruit tissues. Moreover, the expression level of *CmaCAMTA4*, *CmaCAMTA1*, *CmaCAMTA5*, *CmaCAMTA3*, *CmaCAMTA6*, and *CmaCAMTA2* in fruits was higher than that in leaves, indicating that these genes may play important roles in fruits.

### Transcriptional Patterns of *CmoCAMTAs* and *CmaCAMTAs* in Leaf Vein and Leaf Mesophyll Under Salt Stress

**FIGURE 7:**
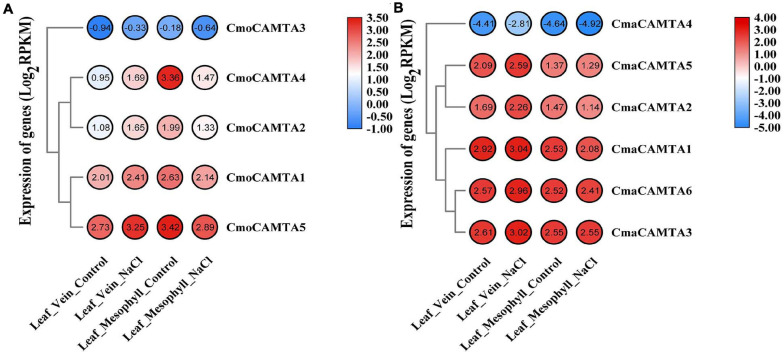
The expression level of the *CAMTA* genes in *C. moschata* (“N15”) and *C. maxima* (“N12”) under salt stress. **(A)** Heat map and hierarchical clustering of five *CmoCAMTAs* in leaf mesophyll and leaf vein under NaCl treatment and control conditions. **(B)** Heat map and hierarchical clustering of six *CmaCAMTA* genes in leaf mesophyll and leaf vein under NaCl treatment and control conditions. All data and the bar on the right of the heat map were standardized by Log_2_ (RPKM).

In *Cucurbita* Linn. plants, to explore the response of *CAMTAs* in leaf veins and leaf mesophyll under salt stress, we analyzed them based on previous RNA-seq data. The results of the heat map and cluster analysis showed that all *CmoCAMTAs* in the leaf vein were significantly induced under salt stress, while all *CmoCAMTAs* in the leaf mesophyll were inhibited under salt stress (Figure 7A). In *C. maxima* (“*N12*”), the expression of these genes was similar to that in *C. moschata* (“*N15*”) (Figure 7B). Overall, the relative expression level of *CAMTAs* in *C. maxima* was higher than that in *C. moschata*. The expression levels of *CmoCAMTA3* and *CmaCAMTA4* were lower than those of other genes in the same cultivar (Figures 7A,B), so it was speculated that *CmoCAMTA3* and *CmaCAMTA4* downregulation may be conserved in *Cucurbita* species under salt stress.

### qRT-PCR Verification of *CmoCAMTAs* and *CmaCAMTAs* in Leaf Vein and Leaf Mesophyll Under Salt Stress

**FIGURE 8:**
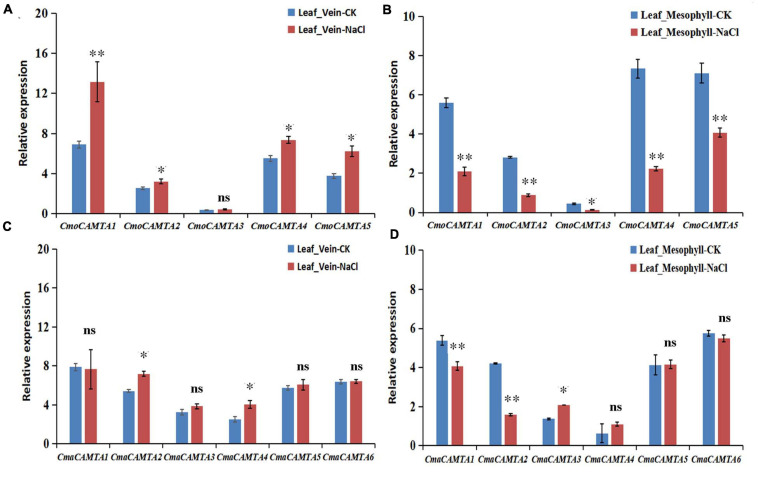
Expression level of the *CAMTA* genes in *C. moschata* (“*Baimi 9*”) and in *C. maxima* (“*Beiguan*”) under salt stress. **(A)** The expression level of the *CAMTA* genes in the leaf vein of “*Baimi 9.*” **(B)** The expression level of the *CAMTA* genes in the mesophyll of “*Baimi 9*.” **(C)** The expression level of the *CAMTA* genes in the leaf vein of “*Beiguan.*” **(D)** The expression level of the *CAMTA* genes in the mesophyll of “*Beiguan*.” The data were calculated by the 2^–ΔΔ*Ct*^ method, and we used the reference gene (β-actin) to correct the expression level of the target gene. Errors bars indicated the standard errors of three biological replicates, and asterisks indicated that the expression levels of genes under CK and salt treatment were significantly different in between (^∗^*P* < 0.05, ^∗∗^*P* < 0.01).

To further determine the response of *CmoCAMTAs* and *CmaCAMTAs* in leaf vein and leaf mesophyll under salt stress, we treated *C. moschata* “*Baimi 9*” and *C. maxima* “*Beiguan*” with a NaCl solution. After 12 h of salt stress, phenotypic observation showed no significant difference between the saline-treated seedlings and the control. However, in the leaf vein of “*Baimi 9*,” the results showed that after 12 h of NaCl treatment, the relative expression profiles of *CmoCAMTA1*, *CmoCAMTA2*, *CmoCAMTA4*, and *CmoCAMTA5* in treated samples were 1.27–1.9 times that of the control samples (Figure 8A). Only *CmoCAMTA3* had no significant difference under salt stress. In the leaf mesophyll of “*Baimi 9*,” the relative expression level of all *CmoCAMTAs* decreased to 30–57% of the control samples under salt stress (Figure 8B). In the vein of “*Beiguan*,” the expression levels of *CmaCAMTA2* and *CmaCAMTA4* under salt stress were 1.32 and 1.61 times that of the control, respectively (Figure 8C). The relative expression levels of *CmaCAMTA1* and *CmaCAMTA2* in the leaf mesophyll under salt stress decreased to 37–52% of the control treatment, while the relative expression levels of *CmaCAMTA3* under salt stress were 1.52 times that of the control treatment (Figure 8D). Comprehensive analysis indicated that *CmoCAMTA1*, *CmoCAMTA2*, *CmoCAMTA4*, *CmoCAMTA5*, and *CmaCAMTA2* played a key role in both veins and mesophyll, *CmaCAMTA5* and *CmaCAMTA6* did not respond to salt stress, and the remaining genes played a role only in veins or mesophyll.

## Discussion

So far, six *CAMTA* genes from *A. thaliana* (Zhang et al., 2019), six *CAMTA* genes from *Gossypium arboreum* (Wei et al., 2017), seven *CAMTA* genes from *Gossypium raimondii* (Wei et al., 2017), nine *CAMTA* genes from *Z. mays* (Yue et al., 2015), 15 *CAMTA* genes from *G. max* (Wang et al., 2015), eight *CAMTA* genes from *P. vulgaris* L. (Büyük et al., 2019), five *CAMTA* genes from *Musa acuminata* (Meer et al., 2019), seven *CAMTA* genes from *S. lycopersicum* (Yang et al., 2012), and eight *CAMTA* genes from *Brassica campestris* ssp. *chinensis* (Hu et al., 2015) have been identified. In this study, a total of 11 predicted *CAMTAs* (five *CmoCAMTAs* and six *CmaCAMTAs*) in *Cucurbita* Linn. were identified using bioinformatics tools.

The identified CmoCAMTA and CmaCAMTA proteins ranged from 914 to 1,089 aa, which was similar to the CAMTA proteins from other different plant species (Wei et al., 2017; Zhang et al., 2019). Except for CmoCAMTA4, CmoCAMTA2, CmaCAMTA5, and CmaCAMTA4, which have higher theoretical isoelectric points (7.21–7.71), the remaining CAMTA proteins all had theoretical isoelectric points of less than 7 (Table 1), indicating that these proteins may be positively charged at a physiological pH. Subcellular localization prediction analysis showed that all CmoCAMTAs and CmaCAMTAs were located in the nucleus, which was consistent with the characteristics of TFs.

To evaluate the evolutionary relationship of *CAMTA* gene families in *Cucurbita* Linn., a total of five CmoCAMTA, six CmaCAMTA, and six AtCAMTA proteins were analyzed. The phylogeny tree showed that these genes were assigned to three subfamilies (Subfamilies I, II, and III) (Figure 1), which was similar to the report of *A. thaliana* CAMTA protein (Zhang et al., 2019). The phylogeny relationships showed that the same subfamily contained CAMTAs from *C. moschata*, *C. maxima*, and *A. thaliana*, indicating that they may come from the same ancestor. In addition, the homology relationship between *C. moschata CAMTAs* and *C. maxima CAMTAs* was closer than that of *A. thaliana* (Figure 1). Based on the phylogenetic tree of *CAMTA* family genes in *C. moschata* and *C. moschata*, five orthologous gene pairs were identified, and they were *CmoCAMTA5*_*CmaCAMTA6*, *CmoCAMTA3*_*CmaCAMTA4*, *CmoCAMTA1*_*CmaCAMTA1*, *CmoCAMTA4*_*CmaCAMTA5*, and *CmoCAMTA2*_*CmaCAMTA2*.

Figure 2 reflected the gene structure of *CAMTAs* in *C. moschata* and *C. maxima*; the exon number of CAMTAs in *Cucurbita* Linn. was between 12 and 14, which was similar to the exon number of CAMTAs in *G. max*, and *Z. mays* (Wang et al., 2015; Yue et al., 2015). It showed that CAMTAs have important conservation among plant species. Orthologous gene pairs usually contain similar intron–exon structures, such as the orthologous gene pair *CmoCAMTA4*_*CmaCAMTA5* and *CmoCAMTA2*_*CmaCAMTA2*, which contained the same number of exons and introns, respectively (Figure 2). Structural analysis showed that all CAMTAs of *C. moschata* and *C. maxima* contained ankyrin repeats, IQ motifs, IPT/TIG domain, and CG-1 DNA-binding domain (Figure 3B and Supplementary Table 2). This result is consistent with previous analysis in the *P. vulgaris* (Büyük et al., 2019), *Z. mays* L. (Yue et al., 2015), and *Fragaria ananassa* (Leng et al., 2015) *CAMTA* gene families. According to previous studies, CAMTAs can be divided into two groups according to the existence of the TIG domain (Rahman et al., 2016b). Based on this classification, *Cucurbita* Linn. CAMTA protein belongs to a class containing TIG, while *A. thaliana* belongs to plants containing non-TIG CAMTA protein (Rahman et al., 2016b).

Chromosome location of *CAMTA* in *Cucurbita* Linn. showed that 11 *CAMTA* genes were unevenly distributed on chromosomes and that three duplicated gene pairs have fragment duplication events, which were known to have occurred between 8.62 and 9.64 MYA. In addition, the ratio of *Ka* to *Ks* of all duplicated gene pairs were less than 1 (Supplementary Table 4), indicating that these gene pairs have undergone purification selection. *Cis*-acting element analysis showed that the duplicated genes pairs *CmoCAMTA4*_*CmoCAMTA2* and *CmaCAMTA5*_*CmaCAMTA2* all contain G-box and ABRE *cis*-acting elements, so it was speculated that these two duplicated gene pairs may have similar functions.

The expression level of *CmaCAMTA4* was lower than that of other genes, which indicated that the expression of *CmaCAMTA4* was limited in time and space. Furthermore, the duplicated genes *CmaCAMTA6*_*CmaCAMTA3* and *CmaCAMTA*5_*CmaCAMTA2* contained similar tissue expression patterns (Figure 6), indicating that these two duplicated gene pairs may have similar functions.

The promoter sequence obtained from the *Cucurbit* genomics database was extracted to detect the *cis*-acting elements of *CAMTA* genes in *Cucurbita* Linn. As a result, many *cis*-acting elements were found, including ABRE, G-box, TGA-element, TGACG-motif, GT1-motif, and MBS (Figure 5). Related studies showed that ABRE, G-box, MBS, GT1-motif, TGACG-motif, and TGA-element had regulatory effects under salt stress (Yamniuk and Vogel, 2004; Saeediazar et al., 2014). Therefore, we hypothesized that *CAMTAs* from *C. moschata* and *C. maxima* played key roles under salt stress. This has a theoretical basis for further studying the function and mechanism of salt-resistant genes.

Studies on *CAMTAs* involved in salt stress response have also been reported in other species. In citrus, three *CsCAMTA* (*CsCAMTA1*, *CsCAMTA3*, and *CsCAMTA5*) genes responded significantly under NaCl treatment (Ouyang et al., 2019). The expression level of *CsCAMTA1* gradually decreased under salt stress and reached the peak at 24 h, which was 3.5 times lower than that of the control group. However, the expression of *CsCAMTA5* and *CsCAMTA3* decreased significantly only 24 h after treatment. In *F. ananassa*, the expression of *FaCAMTA1* and *FaCAMTA4* increased at 2 h and decreased at 12 h. The expression level of *FaCAMTA3* was increasing under salt stress (Leng et al., 2015). Based on the RNA-seq data (BioProject: PRJNA464060) of *Cucurbita* Linn. leaves under salt stress (Niu et al., 2018), the transcription profiles of *CmoCAMTAs* and *CmaCAMTAs* in the leaf vein and leaf mesophyll were also analyzed, and we found that the transcription level of *CmoCAMTA3* and *CmaCAMTA4* was lower than that of other genes. Considering that *CmoCAMTA3* and *CmaCAMTA4* are orthologs, maybe this is the reason why they have the same low expression. At the same time, most of *CmoCAMTAs* and *CmaCAMTAs* played important roles in the vein under salt stress (Figure 7).

To further verify the role of *CmoCAMTAs* and *CmaCAMTAs* in leaf veins and leaf mesophyll under salt stress, we used qRT-PCR technology for further verification (Figure 8). In the leaf vein of “*Baimi 9*,” except from *CmoCAMTA3*, the *CmoCAMTAs* were significantly upregulated by salt stress (Figure 8A). In the mesophyll of “*Baimi 9*,” all *CmoCAMTAs* were significantly inhibited under salt stress (Figure 8B). For “*Beiguan*,” the expressions of *CmaCAMTA2* and *CmaCAMTA4* in leaf veins were significantly upregulated under salt stress, while the expressions of other genes showed no significant differences (Figure 8C). In mesophyll, the expression of *CmaCAMTA1* and *CmaCAMTA2* was significantly decreased under salt stress, while the expression of *CmaCAMTA3* was significantly increased under salt stress, while the expression of the remaining genes showed no significant difference (Figure 8D). This result was consistent to the RNA-seq result, and they showed that *CAMTAs* had different responses to salt stress in the leaf vein and leaf mesophyll. According to the study of Niu et al. (2018), we speculate that this mechanism may be associated with the ability of the tolerant species to accumulate more Na^+^ in the leaf vein and to retain more K^+^ in the leaf mesophyll. Through the analysis of the expression level of *CmoCAMTAs* and *CmaCAMTAs* genes under salt stress in leaf veins and leaf mesophyll, it provides theoretical basis for further exploring the function of *CmoCAMTAs* and *CmaCAMTAs* genes and clarifying the molecular mechanism of these genes and is also of great guiding significance for the cultivation of new varieties of salt resistance.

## Conclusion

In summary, we identified five *CmoCAMTAs* and six *CmaCAMTAs* in the *Cucurbita* genome according to a comprehensive analysis and provided the genetic information of gene distribution, gene structure, protein structure, and evolutionary relationship. We also examined the expression patterns of 11 *CAMTAs* in different tissues. Meanwhile, we explored the responses of five *CmoCAMTAs* and six *CmaCAMTAs* to salt stress, and it is found that these genes offer fundamental clues about their involvement in salt stress.

## Data Availability Statement

The datasets presented in this study can be found in online repositories. The names of the repository/repositories and accession number(s) can be found in the article/Supplementary Material.

## Author Contributions

CS conceived, designed, and analyzed the data. CS and JY wrote the manuscript. BC and XL identified *Cucurbitaceae CAMTAs* and analyzed the gene structure. CS and XL studied chromosome distribution and gene duplication of *Cucurbitaceae CAMTAs*. JY supervised the research. CS, AS, and JY revised the manuscript. All authors have read and approved the manuscript.

## Conflict of Interest

The authors declare that the research was conducted in the absence of any commercial or financial relationships that could be construed as a potential conflict of interest.

## Footnotes

1 http://cucurbitgenomics.org/

2 http://www.ncbi.nlm.nih.gov/

3 https://www.ncbi.nlm.nih.gov/Structure/cdd/wrpsb.cgi

4 http://smart.embl-heidelberg.de/

5 http://web.expasy.org/tools/

6 http://gsds.cbi.Pku.edu.cn/

7 http://meme-suite.org/

8 https://www.ncbi.nlm.nih.gov/Structure/bwrpsb/bwrpsb.cgi

9 http://visualization.ritchielab.psu.edu/home/index

10 http://bioinformatics.psb.ugent.be/webtools/plantcare/html/

## Supplementary Material

Supplementary Figure 1 Detailed information about the LOGOs of the motifs from CmoCAMTA and CmaCAMTA proteins. They were also obtained with MEME.

Supplementary Table 1 The information on coding sequence and protein sequence of CmoCAMTAs and CmaCAMTAs.

Supplementary Table 2 Prediction of the conserved domain position of CmoCAMTAs and CmaCAMTAs.

Supplementary Table 3 List of primer sequences used for the qRT-PCR analysis of *CmoCAMTA* and *CmaCAMTA* genes.

Supplementary Table 4 *Ka*/*Ks* and estimated divergence time for duplicated gene pairs from *CmoCAMTA* and *CmaCAMTA*.

Supplementary Table 5 The position and sequence prediction of salt-related *cis*-acting elements in *CmoCAMTA* and *CmaCAMTA* gene promoters.
