# Supplementary figures and images for: Genome-Wide Characterization and Expression Analysis of CAMTA Gene Family Under Salt Stress in Cucurbita moschata and Cucurbita maxima
Source: Front Genet. 2021 Jun 17;12:647339. doi: 10.3389/fgene.2021.647339 (PMC8249228; doi:10.3389/fgene.2021.647339)

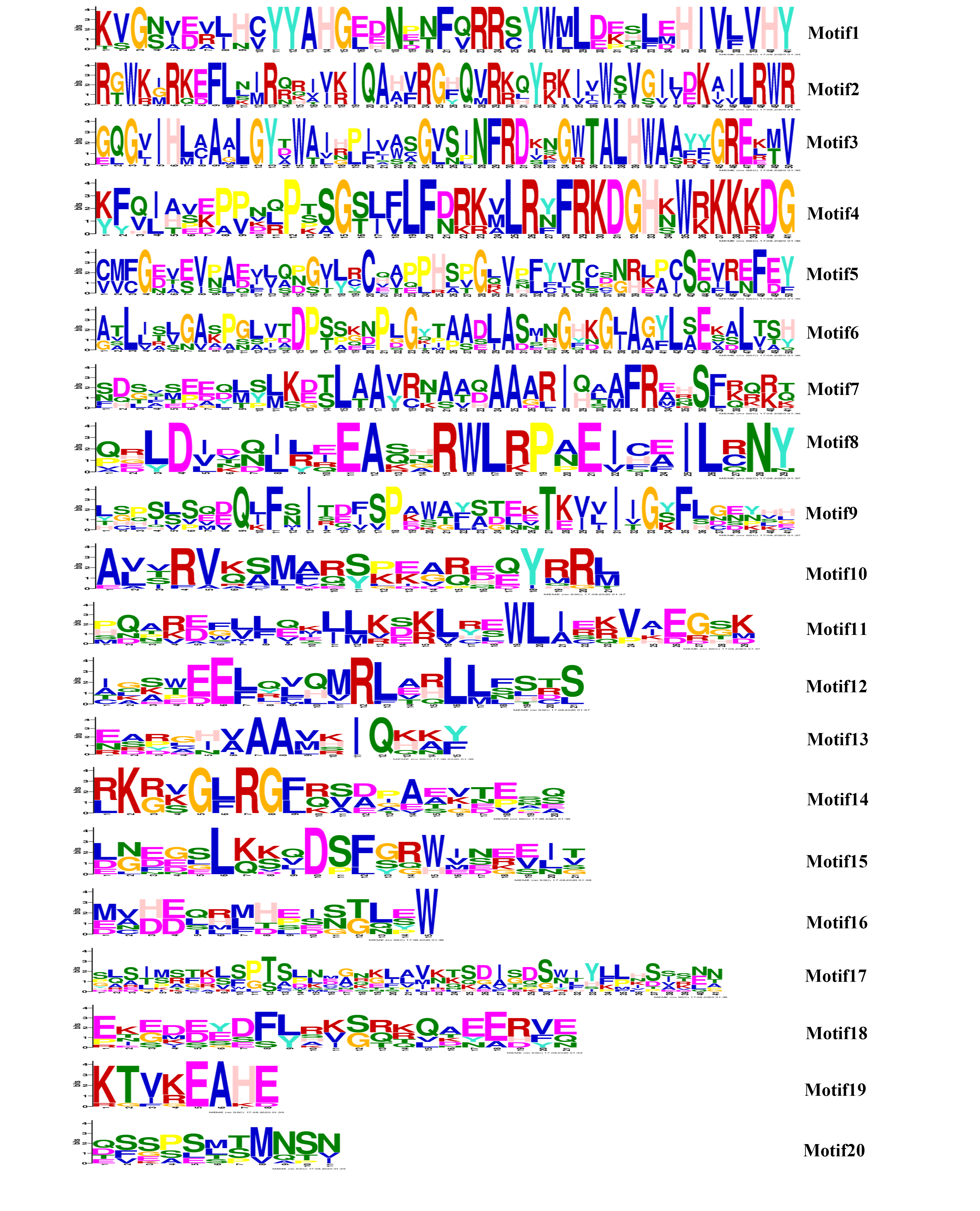

Supplement: Supplementary Figure 1 — Detailed information about the LOGOs of the motifs from CmoCAMTA and CmaCAMTA proteins. They were also obtained with MEME. [file Image_1.JPEG]
